# Palmitoyl-Epigallocatechin Gallate Modulates COX-2-Based Production of Inflammation-Related Oxylipins: Synthesis, Characterization, and Bioevaluation In Vitro and In Silico

**DOI:** 10.1021/acsomega.5c04117

**Published:** 2025-07-29

**Authors:** Concepción Medrano-Padial, Pablo Fuentes-Soriano, Diego Hernández-Prieto, Cristina García-Viguera, Raúl Domínguez-Perles, Carlos Romero-Nieto, Sonia Medina

**Affiliations:** † Laboratorio de Fitoquímica y Alimentos Saludables (LabFAS), 16379 CSIC, CEBAS, Campus Universitario de Espinardo, Edificio 25, Murcia 30100, Spain; ‡ Faculty of Pharmacy, 16733 University of Castilla-La Mancha, Calle Almansa 14 − Edif. Bioincubadora, Albacete 02008, Spain

## Abstract

Lipophenols are esterifications of (poly)­phenols with fatty acids, recently demonstrated to exhibit enhanced bioactivities compared to native phenolics. Among them, catechin lipophenols have been noted as powerful antioxidants present in natural products (e.g., tea leaves), although they remain underexplored. Hence, to analyze the biological advantages of catechin lipophenols, in the form of palmitoyl-epigallocatechin gallate (PEGCG), its de novo synthesis by organic chemistry procedures was implemented. The synthesis product was characterized by LC-MS and NMR. The anti-inflammatory potential was assessed in silico (molecular docking) and in vitro to unravel the PEGCG capacity to prevent inflammation and oxidative stress compared to epigallocatechin gallate (EGCG), shedding light on the biological advantages provided by the lipophilic traits conferred by the fatty acid moiety. PEGCG showed higher ability to inhibit, in vitro, the expression of cyclooxygenase-2 (COX-2) to a greater extent than EGCG (97.03 vs 116.34 ng/mL, respectively). This was further confirmed by retrieving results evidencing lower concentrations of related oxylipins (PGF_2α_, PGE_2_, and 8-iso-PGF_2α_). Interestingly, despite the results concerning the modulation of the COX-2 expression and the higher PEGCG binding affinity against human COX-2 relative to EGCG, as retrieved from the in silico docking assessments, additional verification of EGCG and PEGCG to modulate COX-2 enzymatic activity did not provide conclusive results about their inhibitory capacity, which would require further exploration. These results demonstrate the anti-inflammatory and oxidative stress prevention properties of PEGCG by reducing the COX-2 expression and, beyond the new scientific knowledge provided, obtaining a PEGCG standard will allow an advancement toward identifying new natural sources of lipophenols and their multipurpose applicability as an amphiphilic molecule for functional coproducts.

## Graphical abstract

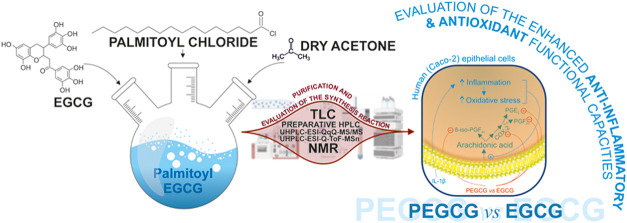

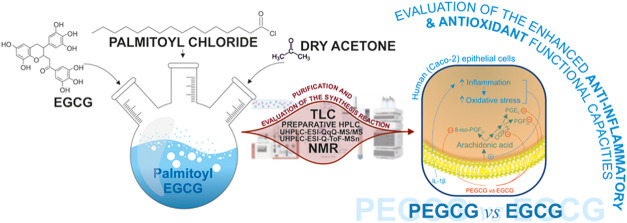

## 1 Introduction

Analyzing food components and approved additives is key to guaranteeing the safety of industrial production and, thus, protecting human health.^1^ This is especially relevant for newly identified families of compounds, namely lipophenols, which result from the esterification of (poly)­phenols with fatty acids. These phenolic derivatives have drawn attention for their potential health benefits as more efficient antioxidants and anti-inflammatory molecules. They are, therefore, pointed out as candidates for applications in treating conditions like neurodegenerative diseases and skin aging. In particular, certain lipophenols have exhibited significant antioxidant activities by increasing proliferation and cytoskeleton remodeling in human keratinocytes, augmenting the scavenging of reactive oxygen species (ROS), and enhancing superoxide dismutase (SOD) enzymatic activity.^2^ Other studies have also demonstrated that (poly)­phenol ester derivatives can inhibit the production of pro-inflammatory mediators and downregulate the expression of inflammatory genes like inducible nitric oxide synthase (iNOS) and cyclooxygenase-2 (COX-2).^3^ According to these bioactivities, they have been evaluated at the neurological level under specific pathophysiological conditions. These evaluations have demonstrated their capacity to incorporate into neuronal membranes, thus suggesting a key role in modulating neurodegenerative pathways in disorders, namely Alzheimer’s disease, among others.^4^ Moreover, these molecules have been stressed by their prominent role in the inhibition of matrix metalloproteinase-9 (MMP-9). In this regard, their cytotoxic character against breast cancer cells has been proven at the in vitro and in vivo scales.^5^ These biological advantages are further boosted by the modification of the lipophilicity/hydrophilicity conferred by the fatty acid moiety. For instance, the esterification of catechins (e.g., epigallocatechin gallate (EGCG)) has demonstrated significant potential to overcome the constraints associated with the hydrophilicity of native (poly)­phenols, whose hydrophilicity compromises bioavailability, reaching operative concentrations in target cells and tissues, and thereby, their functional scope.^6^ Thus, the advantage conferred by the high amphiphilic character of lipophenols lies in their more powerful biological features, since chemical modifications relative to native phenolics enhance their capacity to cross cell membranes and react with molecular targets within cells.^7–10^

When evaluating different plant-based matrices as a source of lipophenols, previous research has demonstrated that, beyond their natural occurrence in plant materials, the formation of these esterified molecules, particularly fatty acid alkyl esters, can change in the plant itself due to specific environmental or stress conditions, and also during food processing or storage, according to the factors and settings involved in industrial procedures.^11^ Meanwhile, the occurrence of catechin lipophenols, specifically palmitoyl-epigallocatechin gallate (PEGCG), in nature has been scarcely described (e.g., in tea leaves).^12^ In this context, obtaining new derivatives through chemical synthesis will be a valuable approach for assessing their biological scope while enabling the investigation of natural sources of these compounds. This is particularly important due to the lack of authentic standards available in the market.^13^ Hence, developing strategies for the esterification of phenolics with acyl or alkyl donors has recently been emphasized as a sustainable method for obtaining new amphiphilic compounds^14,15^ and for enabling the evaluation of the effect of phenolics’ lipophilisation in cells and living organisms, particularly regarding cytotoxicity and enhancement of functional efficiency.

Based on these premises, this study aimed to compare the functional properties of the lipophilized PEGCG relative to EGCG by in silico (molecular docking) and in vitro (intestinal inflammation) models. For that, given the lack of an authentic standard for PEGCG, as a primary activity, a palmitoyl moiety was introduced into the structure of EGCG. Obtaining this lipophenol empowered the assessment of PEGCG concerning the capacity to prevent inflammation and oxidative stress compared to EGCG by monitoring their effects on the concentration and activity of COX-2 and the consequent modulation of the oxylipin cascade (prostaglandins and isoprostanes (PGs and IsoPs, respectively)) in human intestinal epithelial cells (Caco-2).

## 2 Results and Discussion

### 2.1 Combination of LC-MS/MS and NMR for Monitoring the Acylation Reaction Products

As mentioned before, this work evaluated the differential functionality of the palmitoyl derivative of EGCG (PEGCG) concerning inflammation and oxidative stress prevention. To reach this objective, and because of the lack of authentic standards, the target lipophenol should be synthesized and comprehensively characterized as preliminary tasks. To obtain PEGCG, the esterification of EGCG was chemically developed, using palmitic acid chloride, following the methodology described by Liu et al.,^16^ with minor modifications. The process was carried out in a single step, and the efficiency of the synthesis reaction was verified through thin layer chromatography (TLC), which indicated the consumption of the starting EGCG and revealed the formation of constituents with different polarities.

To identify the remaining EGCG and the formation of derivatives during the acylation reaction, the HPLC-ESI-IT-MSn analyses were conducted in the negative ionization mode. The mass spectrometry analyses provided four deprotonated ([M – H]^−^) ions at *m*/*z* 457.28, 915.49, 695.79, and 737.77 arbitrary mass units (amu). All compounds exhibited a maximum absorbance at 274 nm (Figure 1).

**Figure 1:**
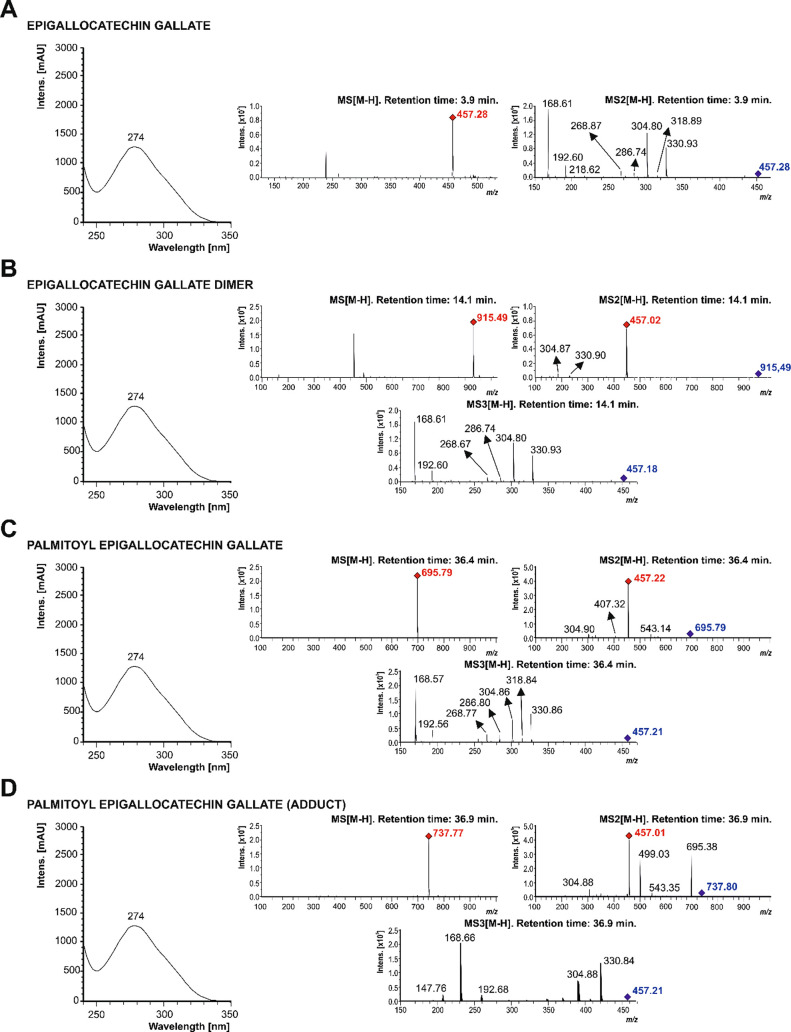
ESI-ion trap mass spectra in negative mode were obtained at the MS, MS2, and MS3 stages of selected ions at *m*/*z* 457.28 arbitrary mass units (amu) for epigallocatechin gallate (A), *m*/*z* 915.49 amu for the epigallocatechin gallate dimer (B), *m*/*z* 695.79 amu for palmitoyl epigallocatechin gallate (C), and *m*/*z* 737.77 amu for the palmitoyl epigallocatechin gallate adduct (D) at the maximum absorption of 274 nm.

Concerning the [M – H]^−^ pseudomolecular ion at *m*/*z* 457.28 amu, the most abundant fragment ions were recorded at *m*/*z* 330.93 and 304.80 amu, corresponding to deprotonated epigallocatechin and gallic acid, respectively (Figure 1A). Moreover, the MS2 fragmentation evidenced the loss of a water molecule [M – H-152–18]^−^ to yield a deprotonated ion at *m*/*z* 286.74 amu, and the presence of the base peak at *m*/*z* 168.61 amu, the most common deprotonated fragment ion corresponding to gallic acid (Figure 1A). The presence of EGCG corresponded to catechin molecules not consumed during the acylation reaction.

Furthermore, fragmentation yielded other high-mass deprotonated ions at very low abundance. The deprotonated ion at *m*/*z* 915.49 amu was deduced based on the assumption of dimerization of two EGCG molecules [2M – H]^−^, with characteristic fragmentation ions and a base peak (100% abundance) at *m*/*z* 457.02 amu, resulting from the dimer dissociation [M – H-457]^−^ that corresponded to EGCG, according to the characteristic fragmentation pattern obtained in MS3 (Figure 1B). This fragmentation pattern agrees with the product ions described in Figure 1A and information previously reported on identifying the EGCG dimer in *Camellia sinensis* tea using UHPLC-PDA-ESI-MS.^17^ Beyond the acylation reaction, this peak was also identified at a low concentration in the solution of authentic standard EGCG (freshly prepared at a micromolar concentration). These findings agree with previous reports applying matching conditions.^16,18–20^ Tentatively, the dimerization of EGCG, which is tuned by several factors, namely temperature, concentration of the native catechin, pH, and the presence of other molecules,^21^ would be due to the rapid degradation of catechins, giving rise to the loss of hydrogen atoms via the polymerization of EGCG because of its auto-oxidation potential.

As a direct result of the EGCG acylation reaction, a deprotonated ion at *m*/*z* 695.79 amu (Figure 1C), identified as EGCG monopalmitate (PEGCG), exhibited a fragmentation pattern governed by a base peak at *m*/*z* 457.22 amu, corresponding to EGCG, which represented the loss of the palmitoyl moiety [M – H-palmitoyl]^−^.^22^ Results from the two stages of tandem mass spectrometry (MS3 analysis) for the unequivocal identification of PEGCG evidenced the fragmentation of the *m*/*z* 457.22 amu ion, rendering the characteristic EGCG pattern (*m*/*z* 330.86, 318.84, 304.86, 286.80, 268.77, 192.56, and 168.57 (100%, base peak) amu) (Figure 1C).

Based on these results, it was concluded that, during the acylation reaction, the PEGCG monoester was formed successfully, while no esters of higher degrees (di-, tri-, or tetra-esters) were generated, tentatively due to the lower solubility of higher-degree esters in acetone.^16^ Beyond the differential polarity of higher-degree esters, their formation could be impaired by the steric hindrance of the long chain of palmitic acid, which might cause difficulty in the acylation process, as stated by Zhong et al.^15^ In this context, the formation of the predominant ester depends on the selectivity and specificity of the acylation reaction, which primarily determines the chemical structure of the parent (poly)­phenolic molecule.^23^

Finally, a deprotonated ion was recorded at *m*/*z* 737.77 amu, corresponding to a PEGCG molecule with a *m*/*z* 42 amu adduct. Accordingly, for the MS2 and MS3 determinations, this compound yielded base peaks at *m*/*z* 457.01 and 168.66 amu, respectively, which match the EGCG and gallic acid deprotonated ions, correspondingly (Figure 1D). The formation of this adduct may be due to the use of sodium acetate in the acylation reaction or acetonitrile as the chromatographic mobile phase B. A previous methodology for synthesizing PEGCG derived from natural tea polyphenols also detected a molecular ion peak at *m*/*z* 737.20, but with a different fragmentation pattern, which was not identified.^16^

### 2.2 Chemical Characterization of Purified PEGCG Using LC-MS/MS and NMR

Identifying PEGCG, formed during the acylation reactions, leads to purification of this lipophenol by preparative HPLC. For this, the crude product of the acylation reaction was dissolved in ACN/LC-MS water (80:20, *v/v*) for the process-scale purification workflow. The preliminary assessment of the PEGCG solutions at 275 nm indicated that the best separation is achieved using a flow rate of 1.0 mL/min, with the column maintained at RT. These conditions allowed the collection of the appropriately separated target PEGCG, further purified by evaporating the solvents. The specific structure of PEGCG in the final oily product was determined using MS (QToF and QqQ) detection systems, operated in the positive and negative ionization modes, respectively, and by ^1^H NMR.

Combining QToF and QqQ MS detection systems constitutes an efficient routine to comprehensively identify unknown or newly synthesized compounds, since they provide complementary information.

The application of such an analytical approach to the purified PEGCG solution showed a base peak chromatogram (UHPLC-QToF-MS/MS) with a single peak corresponding to the purified PEGCG at 2.80 min (Figure 2A). Hence, the monopalmitate of EGCG was identified according to its accurate MS (*m*/*z* 697.3075 amu), which exhibited a mass error lower than 5 ppm, as well as fragment ions at *m*/*z* 459.0824 and 278.2426 amu that agreed with the information reported in the literature.^12,16,22,24^ Moreover, the characterization of the purified PEGCG by UPLC-ESI-QqQ-MS/MS, operated in the multiple reaction monitoring (MRM) mode, allowed the selective identification and quantification of the targeted lipophenol via fast screening of specific precursor ion-to-product ion transitions (*m*/*z*). Upon this analysis, when assessing the synthesis solution by UPLC-ESI-QqQ-MS/MS, PEGCG exhibited the best response in the negative MRM mode. The nebulizer pressure (30 psi), gas flow (8 L/min), gas temperature (325 °C), and two specific MRM parameters (fragmentor voltage (90 V) and collision energy (9 eV)) were optimized to obtain the maximum signal for the quantification precursor/product ion transition selected. The deprotonated pseudomolecular ion [M – H]^−^ of PEGCG (*m*/*z* 695.5 amu) eluted at 3.01, and the precursor > product ion transitions selected for quantification and confirmation were *m*/*z* 695.5 > 407.1 amu and *m*/*z* 695.5 > 457.9 amu, respectively (Figure 2B).

**Figure 2:**
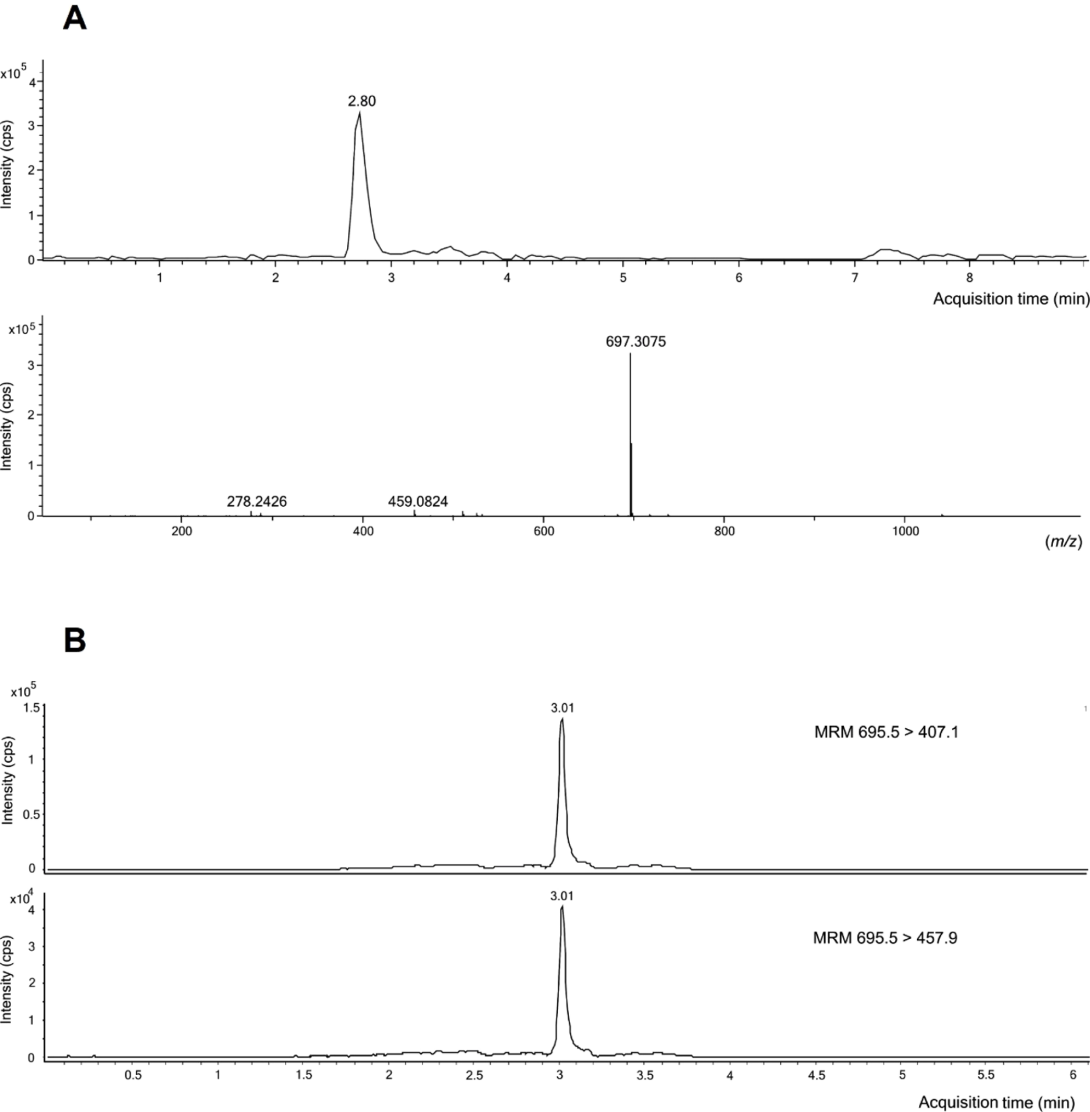
LC-MS Q-TOF analysis of purified PEGCG in ESI positive mode, and mass spectra recorded at *m*/*z* 697.3075 arbitrary mass units (amu). (A) LC-MS/MS chromatogram of the synthesized PEGCG obtained on a triple quadrupole employing dynamic multiple reaction monitoring (MRM) quantification (*m*/*z* 695.5 > 407.1 amu) and confirmation (*m*/*z* 695.5 > 457.9 amu) transitions in ESI negative mode (B).

The ^1^H and ^13^C NMR characterization of PEGCG (Supplementary Figure 1) provided essential complementary information for determining the lipophenol structure. In this regard, the NMR results obtained were consistent with previously reported data for PEGCG,^16^ providing further support for the LC-MS characterization performed, thus allowing a robust setting of the compound’s identity.

### 2.3 Cytotoxic Effect of Palmitoyl-Epigallocatechin Gallate on Caco-2 Cells

Nonaddressed harmful effects must be discarded as a prerequisite for assessing new compounds on their bioactive capacity. In this regard, since the main administration route envisaged for EGCG and PEGCG is dietary, these compounds were tested for their cytotoxic effect on the intestinal epithelium. Thus, the viability of Caco-2 cells was assessed by exposing the cells to decreasing concentrations of EGCG and PEGCG (from 1.000 to 0.001 μmol/L) for 24 and 48 h, while monitoring the trypan-blue exclusion capacity (a characteristic of live cells). As a main result, no significant reduction in the cells’ viability was induced by both catechin derivatives, relative to the untreated control. This outcome was consistent with the absence of cytotoxic effects (Figure 3), in line with previous results on the safety of other lipophenols.^25^ Beyond this, evidence gathered to date shows that the structural modifications featuring lipophenols enhance cell viability and proliferation capacity by contributing to the cell membrane integrity. This activity depends on the alkyl chain length and the lipid moiety’s unsaturation degree.^7,25^ This is because the capacity to cross the cell membrane through more efficient associations depends on the structural traits of the lipid chain, which, beyond enhancing access into cells, confers protection against oxidation and inhibits the formation of toxic aldehydes.^26,27^ This protective activity would preserve the fluidity of the cell membrane,^7^ thereby giving lipophenols the capacity to boost cell proliferation and functionality.^28^

**Figure 3:**
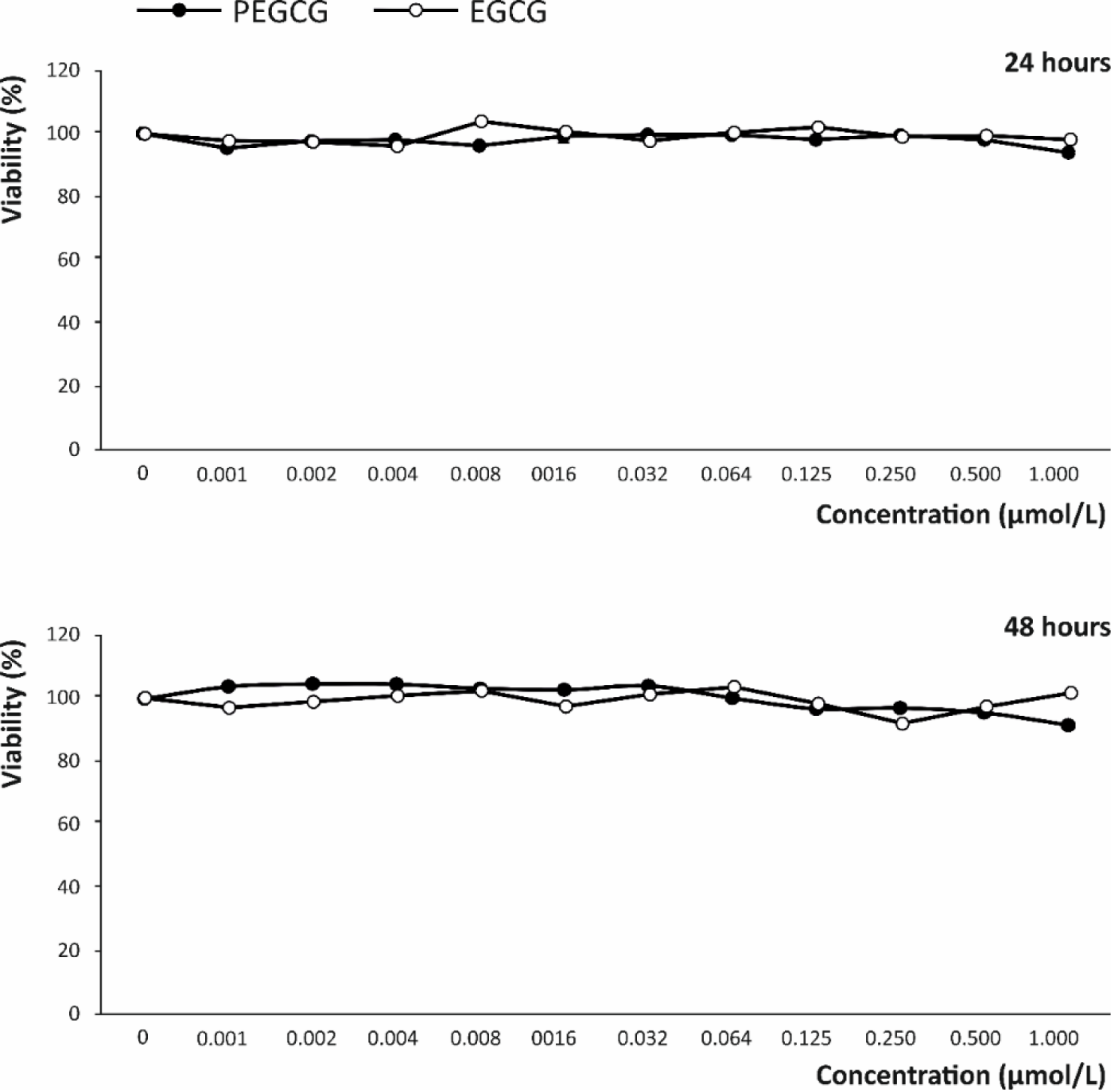
Cytotoxicity on Caco-2 cells (intestinal epithelial line) at decreasing concentrations of EGCG and PEGCG, ranging from 1.000 to 0.001 μmol/L, measured by a trypan blue exclusion assay during the logarithmic growth phase of Caco-2 cells. Cytotoxicity is expressed as the mean ± SD (*n* = 3) of the viability percentage calculated relative to the untreated control at 24 and 48 h. Results are representative of three independent experiments.

These biological benefits, together with the physical properties described for PEGCG (e.g., greater thermal stability and resistance to the alkaline conditions featuring the intestinal phase of digestion, in comparison with EGCG,^16^ encourage the comparative assessment of PEGCG on the health-related bioactivities associated with inflammation and oxidative stress.

### 2.4 Structure-Based Virtual Screening by Molecular Docking Analysis

In silico modeling of the functional properties of PEGCG relative to its native phenolic counterpart (EGCG) was developed by molecular docking simulations. This modeling provided a ranking of the feasibility of diverse protein–ligand poses and the binding affinity values, considering both phenolic derivatives as ligands with COX-2,^29^ thus shedding light on the modulation of COX-2 activity.

As demonstrated by the affinity calculations retrieved upon GNINA software-based analyses (Table 1), which are based on the conformation proposed by DiffDock, it is evident that COX-2 presented a higher affinity for PEGCG than for EGCG.

**Table 1:** Results Obtained by Molecular Docking Analyses with EGCG and PEGCG as Ligands and COX-2 as the Receptor, Grouped by the Top Three Ranked Conformations Proposed by DiffDock and for Each Conformation, Values for the Top Three Calculations Delivered by GNINA

Ligand	Binding energies (kcal/mol)	CNN score	CNN affinity (ln(M^–1^))
EGCG—rank1	1: −8.10	0.9096	5.321
	2: −9.06	0.8042	5.548
	3: −9.46	0.7994	5.768
PEGCG—rank1	1: −9.50	0.9624	8.911
	2: −8.68	0.9138	8.730
	3: −8.32	0.8756	8.202
EGCG—rank2	1: −10.11	0.9186	6.129
	2: −8.81	0.8305	5.664
	3: −7.97	0.8181	5.241
—PEGCG—rank2	1: −9.31	0.9675	9.287
	2: −9.54	0.9301	9.471
	3: −8.83	0.9150	9.345
EGCG—rank3	1: −10.58	0.8464	6.179
	2: −7.24	0.7223	5.164
	3: −7.63	0.6651	4.773
PEGCG—rank3	1: −9.32	0.9632	9.629
	2: −9.31	0.9477	9.231
	3: −9.00	0.8507	8.894

Despite the binding energies of the first conformation of EGCG for the rank 2 and 3 locations provided by DiffDock being lower than those of PEGCG (−10.11 and −10.58, respectively), their CNNScore was lower than almost all the CNNScores for PEGCG (0.9186 and 0.8464). This indicated that the binding energy of EGCG has a higher uncertainty than the PEGCG calculations (the average CNNScore for PEGCG was 0.925). Moreover, the CNN affinity for those cases, a logarithm of the binding constant, was 6.129 and 6.179. These represented 2 orders of magnitude less in the binding constant than the values for PEGCG. Therefore, PEGCG might play a more powerful role in the COX-2 regulating activity and the subsequent production of oxylipins, which, in the present study, has been determined by resorting to the Caco-2-based intestinal model (as described below). Additionally, further insights were provided by a visual 3D representation of the molecular docking simulation, such as the stability provided by the palmitoyl moiety to PEGCG concerning an enhanced attachment to COX-2 (Supplementary Figure 2). Furthermore, the robustness of the PEGCG proposed poses was evidenced by comparison with the EGCG ones, since the EGCG had scarce consensus among their different poses.

Although upon in silico studies, in concordance with this work, certain phenolics such as catechins are featured by high affinity for COX-2,^30^ their impact on enzyme activity remains inconclusive. Therefore, further investigations are warranted to explore the structure–activity relationship of the COX-2 expression and activity and the precise mechanisms underlying the observed anti-inflammatory activity.

### 2.5 Inhibitory Effect of Palmitoyl-Epigallocatechin Gallate on Cyclooxygenase-2 Expression and Activity

Despite promising in silico affinities, experimental data have yielded variable results regarding the inhibitory effects of EGCG and PEGCG on the COX-2 expression and activity. Thus, molecular docking provided estimated binding affinities of EGCG and PEGCG against COX-2, which should be further supported by the in vitro analysis of the COX-2 expression in cells exposed to the bioactive lipophenols (EGCG and PEGCG) and inhibition of activity. In this regard, the fact that IL-1β and COX-2 play a central role in inflammatory processes is nowadays broadly demonstrated. Hence, a diversity of in vitro models of gastrointestinal inflammation have demonstrated a significant augmentation of COX-2 expression secondary to IL-1β-induced inflammation. This increase induces a subsequent augmented production of the putative isoprostanoids in close association with inflammation and oxidative stress,^28,31^ since they act as biomarkers of such biological processes.^32^

Because of the key role of COX-2 in the onset and evolution of the inflammatory process, identifying new bioactive compounds capable of modulating its levels and fine-tuning the inflammation-related isoprostanoids is of remarkable interest. Indeed, these molecular tools modulate the course of inflammation and prevent a subsequent loss of functionality. In this context, the present study assessed PEGCG concerning its capacity to downregulate the COX-2 expression in Caco-2 cells under IL-1β-related inflammatory conditions, and its enzymatic activity.

Caco-2 cells exposed to the proinflammatory stimulus, without the concourse of the modulators under study (PEGCG and EGCG), augmented by 2.4-fold the concentration of COX-2, up to 136.79 ng/mL, from the level in untreated cells to 56.86 ng/mL (negative control) (Figure 4). Meanwhile, although both EGCG and PEGCG partially prevented the augmentation of the COX-2 induced by IL-1β, the lowering capacity was only significant (*p* < 0.05) for PEGCG (97.03 ng/mL). On the contrary, the native catechin (EGCG) was not able to significantly reduce the IL-1β-induced increase in COX-2 (116.34 vs 136.79 ng/mL, respectively) (Figure 4).

**Figure 4:**
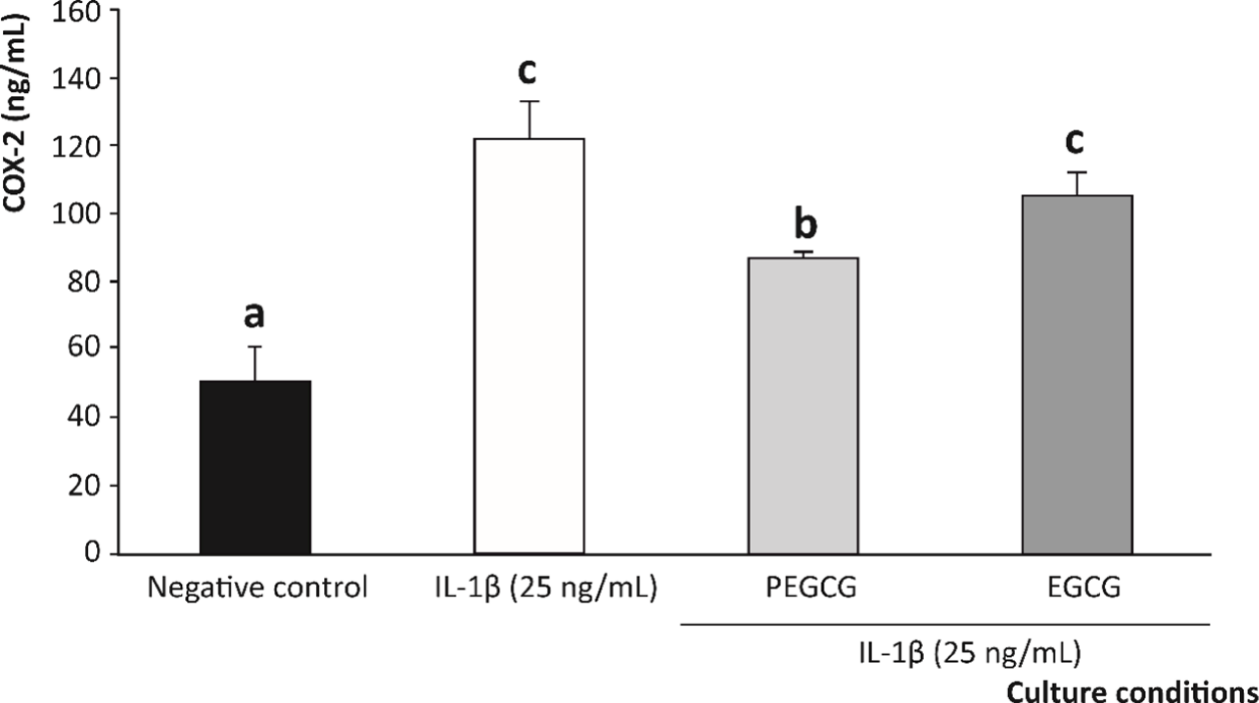
Modulation of the cyclooxygenase-2 (COX-2) concentration (ng/mL) after 10 h of exposure to IL-1β (25 ng/mL) by PEGCG and EGCG. Concentration is expressed as mean ± SD (*n* = 3). Distinct lowercase letters indicate values significantly different at *p* < 0.05, according to one-way analysis of variance (ANOVA) and Tukey’s multiple range test.

To date, it has been suggested that the higher lipophilicity of PEGCG compared with EGCG, conferred by the lipid moiety, provides biochemical and biological advantages. Specifically, the lipophilic trait of PEGCG increases the ability of EGCG to integrate into the lipid bilayers and thus can act to prevent lipid peroxidation and the formation of toxic radicals more efficiently.^24,33^ This would be reflected in an attenuated production of mediators of inflammation and oxidative stress catalyzed by COX-2.^34^ Nonetheless, the biological power of lipophenols seems to be multifactorial, depending not only on lipophilicity but also on the concentration achieved in the different cell compartments and the membrane receptor-binding capacity. In this regard, the dose used in the current study (1.000 μmol/L for both PEGCG and EGCG) provides more robust evidence of the actual biological scope relative to the in vivo conditions, in comparison with previous descriptions of EGCG ester derivatives tested by applying supraphysiological doses (up to 40 μM). Indeed, this concentration might cause harmful effects on cell viability and thus skew the conclusions on the actual biological interest of lipophenols.^15^

When reviewing the enhanced capacity of PEGCG in inhibiting COX-2, it was hypothesized that this might be due to its ability to downregulate the expression of COX-2-encoding genes at the transcriptional level, mediated by the redox-sensitive nuclear factor κ-light-chain-enhancer of activated B cells (NF-κB) and activator protein-1 (AP-1).^34^ Indeed, this is a common mechanism of action for many antioxidants, including EGCG and its ester derivatives, which entail significant anti-inflammatory properties. In this context, a previous study highlighted the antioxidant potential of another lipophenol (EGCG-DPA), which alleviated oxidative stress by inhibiting the activation of the translocation factors NF-κB and AP-1 and regulating the expression of COX-2-encoding genes.^15^ In this connection, the present study goes beyond by analyzing how the lipid moiety of PEGCG might allow the development of anti-inflammatory and antioxidant activities more efficiently than EGCG by lowering the synthesis of several COX-2-derived arachidonic acid isoprostanoids (PGs and IsoPs) and analyzing its capacity to reduce the enzymatic activity of COX-2 (especially because of the binding activity demonstrated by resorting to molecular docking assessments). This approach suggested that the binding affinity does not always correlate with an operative capacity to inhibit enzyme activity. In this context, the COX-2-mediated anti-inflammatory activity reported in the present work seems to be related to a decreasing effect on COX-2 expression. At the same time, the main results retrieved indicated that EGCG and PEGCG did not significantly inhibit COX-2 activity (data not shown). In line with these findings, previous studies have suggested that phenolic compounds (e.g., EGCG) can prevent inflammation by reducing COX-2 expression without affecting its enzymatic activity, primarily through mechanisms involving the modulation of transcription factors like NF-κB and pathways such as mitogen-activated protein kinase (MAPK) and phosphatidylinositol 3-kinase/protein kinase B (PI3K/Akt). Moreover, it has been reported that the anti-inflammatory effects of EGCG are based on its capacity to inhibit the transfection of NF-κB and Activator Protein-1 (AP-1) thereby downregulating the expression of iNOS and COX-2. This occurs mainly by scavenging NO, peroxynitrite, and other reactive oxygen species (ROS)/reactive nitrogen species (RNS) and thus, decreases the production of inflammatory factors.^35^

Summarizing, while the theoretical binding affinity predicted by computational models performed in this study suggested interactions between EGCG/PEGCG and COX-2, and thus a potential inhibitory activity, these interactions did not consistently inhibit the enzyme’s activity. This discrepancy underscores the complexity of enzyme-ligand interactions, suggesting that the prevention of the inflammatory process dependent on COX-2 by PEGCG seems limited to the lipophenol’s capacity to modulate the expression of the enzyme. Accordingly, the necessity for comprehensive experimental validations to fully understand the anti-inflammatory potential of bioactive compounds, which may vary depending on the compound’s chemical structure and the experimental context, is highlighted.

### 2.6 Modulation of COX-2-Related Inflammatory and Oxidative Stress Mediators by Palmitoyl-Epigallocatechin Gallate in Intestinal Inflammation

Despite the lack of EGCG and PEGCG capacity to inhibit the enzymatic activity of COX-2, the extent to which inhibiting the expression of COX-2 by PEGCG relative to EGCG could be enough for lowering the synthesis of inflammation (PGE_2_ and PGF_2α_) and oxidative stress (8-Iso-PGF_2α_) mediators was explored in vitro using a model of intestinal inflammation based on a Caco-2 cell monolayer exposed to pro-inflammatory IL-1β. Prostaglandins and IsoPs are bioactive molecules in the cell membrane derived from arachidonic acid (AA). In particular, PGs have been demonstrated to play a critical role in maintaining the functionality of the epithelial barrier and its characteristic paracellular permeability.^36^ Beyond this role, AA-derived COX-2 mediators and ROS have been proposed as key metabolites defining the clinical course of intestinal disorders.^37^

As shown in Figure 5, the concentration of PGF_2α_ increased in IL-1β-stimulated cells by 1.6-fold, on average, up to 125.90 ng/mL, providing values 55.8% higher than those recorded in the negative control samples (80.80 ng/mL) (Figure 5A). When cells were pretreated with 1.000 μmol/L PEGCG, more efficient inhibition of the PGF_2α_ synthesis was observed relative to the functionality recorded for EGCG, thus providing, the former, a PGF_2α_ concentration of 98.20 ng/mL, close to the values exhibited by the negative control, while EGCG did not significantly reduce PGF_2α_ synthesis (Figure 5A). This result agrees with the enhanced capacity of PEGCG to lower PGF_2α_ due to the higher lipophilicity conferred by the lipid moiety.^24,33^ This chemical trait would also augment its incorporation into the cell membrane relative to EGCG.^26,27^ This would agree with reports concerning lipophenols of quercetin, including the chemical structure of linoleic and α-linolenic acid-based moieties, which upgraded the biological scope of native flavonols.^7^

**Figure 5:**
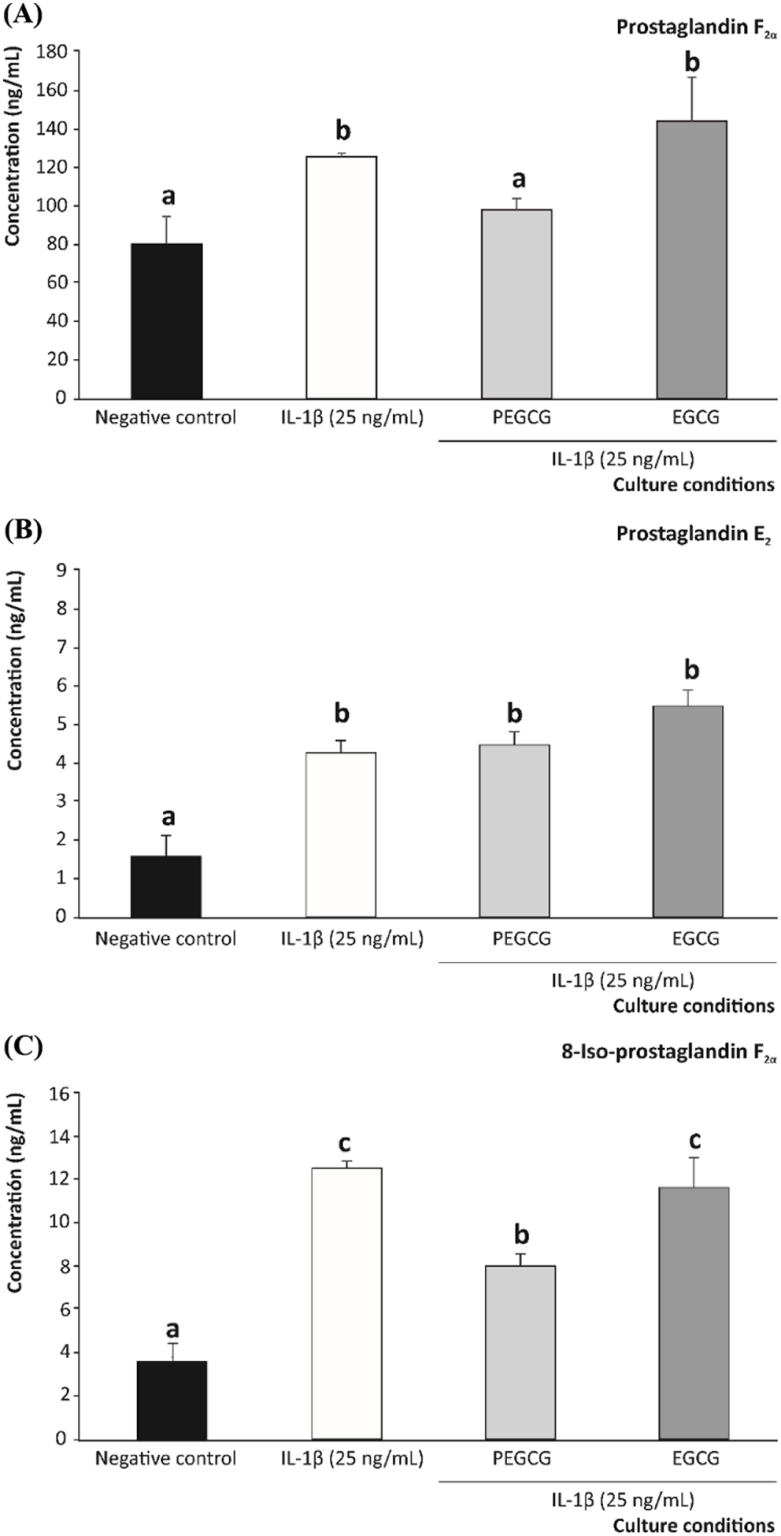
Modulation of oxylipin concentration (ng/mL): PGF_2α_ (A), PGE_2_ (B), and 8-iso-PGF_2α_ (C), after 10 h of exposure to IL-1β (25 ng/mL) by PEGCG and EGCG. Concentration is expressed as mean ± SD (*n* = 3). Distinct lowercase letters indicate values significantly different at *p* < 0.05 (*) according to one-way analysis of variance (ANOVA) and Tukey’s multiple range test.

Concerning PGE_2_, this inflammatory mediator is featured by triggering pleiotropic effects on cell physiology, depending on the specific receptor to which it binds (EP 1–4).^38^ As a result, PGE_2_ modulates the inflammatory process by acting as a putative inflammation mediator (pro-inflammatory and anti-inflammatory), resulting in a complex interaction network involving factors such as time, concentration, and culture conditions.^39^ In the present work, the level of PGE_2_ expressed by unstimulated Caco-2 cells (1.74 ng/mL) increased 2.4-fold, on average, under pro-inflammatory conditions (4.14 ng/mL). In contrast to the behavior observed for PGF_2α_, the PGE_2_ augment induced by IL-1β was not significantly prevented by either PEGCG or EGCG (*p* > 0.05) (Figure 5B). In this context, a previous study described the efficient anti-inflammatory activity of EGCG esterified with docosapentaenoic acid (DPA), mediated by a more efficient inhibition of PGE_2_ than the unesterified DPA. Nonetheless, the operative concentrations tested in this work (from 25 to 50 μg/mL) were much higher than the physiological concentrations that could be achieved in vivo and applied.^14^

Beyond the markers referred to, as a side effect, inflammation entails an augmented cell metabolism and the production of harmful ROS amounts that entail deleterious effects on critical molecules (e.g., lipid peroxidation or alteration of the nucleotide sequence in the DNA).^40^ This enhanced oxidative stress is mirrored by increased IsoPs, mainly represented by augmented concentrations of 8-iso-PGF_2α_ (15-F_2t_-Isoprostane).^41^ Hence, exposing Caco-2 cells to pro-inflammatory IL-1β raised the 8-iso-PGF_2α_ concentration from 3.58 ng/mL (negative control) to 12.40 ng/mL (pro-inflammatory environment). Interestingly, cells pretreated with PEGCG significantly decreased the 8-iso-PGF_2α_ concentration achieved under inflammatory conditions by 1.6-fold, which was not observed when treating cells with EGCG (Figure 5C).

The relevance of these results is enclosed in the biology of IsoPs, which are mainly generated through the free-radical-dependent process of lipid peroxidation reactions. Beyond this pathway, the synthesis of IsoPs may be enzymatically catalyzed by an array of enzymes, including COX-2, resulting in excessive oxidative stress secondary to inflammation.^36^ When the efficiency of PEGCG and EGCG in preventing oxidative stress was compared, it was found that PEGCG inhibited the 8-iso-PGF_2α_ production to a greater extent than the native catechin, thus suggesting an enhanced antioxidant capacity in complex biological systems. Specifically related to the antioxidant activity of lipophenols, to date, in vitro models have demonstrated a parabolic behavior, known as the “cutoff” effect. This is characterized by an initial enhancement of the antioxidant efficiency as the alkyl chain increases, up to a critical length.^42^ However, current knowledge on the contribution of the alkyl chain length to improved antioxidant functions of ester derivatives of phenolics is scarce. This gap has been addressed by several studies that synthesized lipophilic derivatives of phenolics through acylation with fatty acids. This approach has highlighted the relevance of the chain length to achieving optimal functionality. In this context, long-chain polyunsaturated fatty acids (C18–C22) enhance the peroxyl radical scavenging and metal chelation capacities of the native EGCG.^14^ Similarly, other authors have studied fatty acid esters of EGCG, incorporating different chain lengths (from C13 to C22), describing excellent antioxidant activities and protective effects in various food model systems.^24,43^ In this regard, it has to be noted that these studies evaluated the antioxidant capacity by spectrophotometric methods (e.g., DPPH, ORAC, and ABTS), which involve significant constraints and do not provide robust evidence. Thus, focusing on biological process markers, such as IsoP, as conducted in the present study, in vitro, provides a deeper understanding of the enhanced bioactivity of lipophilic molecules.

## 3 Conclusions

This study highlights the importance of a lipophilic catechin derivative (PEGCG) as a modulator of inflammation and oxidative stress to a greater extent than the native EGCG. In this context, implementing a chemical synthesis method has allowed obtaining an authentic standard of PEGCG, unavailable on the market, leading to the characterization of the enhanced relevance of its functional traits, conferred by the lipid moiety, regarding inflammation. The application of molecular docking tools predicts the binding capacity of EGCG vs PEGCG to the human COX-2 enzyme. The in vitro assessment of both compounds provided evidence of the enhanced capacity of PEGCG to prevent inflammation and oxidative stress by modulating the expression of COX-2 and the derived isoprostanoids. Nonetheless, both failed in modulating COX-2 enzymatic activity, thus stressing the multifactorial and variable character of enzyme-ligand interactions. According to the lack of inhibitory capacity on COX-2 enzymatic activity, the anti-inflammatory power demonstrated for PEGCG seems to be confined to the potential inhibition of the enzyme’s expression. In addition to these mechanistic facts, the enhanced biological scope attributable to PEGCG would be attributed to improved cellular uptake and membrane incorporation of lipophenol. These findings suggest that PEGCG (representing a diversity of lipophenols) may serve as an alternative to hydrophilic phenolics of interest as cost-effective agents or coadjuvants in treating several pathological processes associated with inflammation and oxidative stress. Moreover, considering the improved biological functions observed in the present study for ester derivatives with amphiphilic character, two main challenges should be addressed in the next years; first, identifying lipophenolic molecules in additional natural matrices by utilizing the newly synthesized standards (even obtaining new standards for a range of lipophenols with tentatively complementary activity and distribution in different plant-based foods and materials) will enable applying the newly discovered sources as functional ingredients and nutraceuticals. Second, augmenting the diversity and scope of the experimental approaches applied to retrieve evidence on the biological interest of bioactive compounds is essential to set up the actual enhanced capacity of these compounds to reach operative concentrations in the different cell types and tissues, as well as maintain their already demonstrated biological efficacy in more complex biological systems (in vivo upon preclinical and clinical assays), which further boost the investigation of new sources and applications of these amphiphilic compounds.

## 4 Materials and Methods

### 4.1 Chemicals and Reagents

(−)-Epigallocatechin gallate (EGCG) (No. CAS 989–51–5, purity 95%), palmitoyl chloride (purity 98%), Bis–Tris (bis­(2-hydroxyethyl)-amino-tris­(hydroxymethyl)­methane), the enzyme β-glucuronidase-sulfatase from *Helix pomatia* type H2 (G0876), l-glutamine, Celite 545, sodium acetate anhydrous, and sodium formate were obtained from Sigma–Aldrich (St. Louis, MO, USA). The authentic standards of oxylipins PGF_2α_, PGE_2_, and 8-iso-PGF_2α_ were purchased from Cayman Chemicals (Ann Arbor, Michigan, USA). All LC-MS grade solvents (acetonitrile, acetone, methanol, and formic acid) were supplied by J.T. Baker (Phillipsburg, NJ, USA). Water was treated using a Milli-Q water purification system from Millipore (Bedford, MA, USA). Thin layer chromatography (TLC) polyester sheets with a silica gel layer (item number: 805021) were acquired from Macherey-Nagel (Düren, Germany). Acetone-*d*_6_ was obtained from Deutero GmbH (Kastellaun, Germany). The solid-phase extraction (SPE) cartridges used were Strata X-AW cartridges (100 mg/3 mL), which were acquired from Phenomenex (Torrance, CA, USA). The human colon adenocarcinoma (Caco-2) cell line (ATCC HTB-37) was obtained from the American Type Culture Collection (ATCC, Rockville, MD, USA). Trypsin-ethylenediaminetetraacetic acid, Eagle’s minimum essential medium (EMEM), l-glutamine, fetal bovine serum, penicillin/streptomycin, and essential amino acids were obtained from Gibco (Thermo Fisher Scientific, Madrid, Spain), and the 24-well plates were obtained from Corning (New York, NY, USA).

### 4.2 Synthesis of Palmitoyl-Epigallocatechin Gallate

The chemical lipophilization of EGCG with palmitoyl chloride to obtain the ester derivative PEGCG was performed following the methodology described in the literature,^16,24^ with minor modifications. Briefly, EGCG (100 mg) was added to 2.20 mL of dry acetone. After complete dissolution, anhydrous sodium acetate and a 1.05 molar ratio of palmitoyl chloride were added dropwise under an N_2_ atmosphere with mechanical stirring at 900 rpm, in the dark, and at room temperature. The reaction mixture was stirred for 5 h and monitored by TLC (petroleum ether/ethyl acetate/acetic acid (1.50:1.00:0.05, *v*/*v*/*v*) to confirm the formation of PEGCG and to check the progress of the chemical reaction through the consumption of EGCG. After reacting for 5 h, the reaction mixture was filtered through Celite 545 (Sigma-Aldrich, St. Louis, MO, USA), washed with 2.00 mL of acetone, and concentrated under reduced pressure until a faint yellow oily product was obtained.

### 4.3 Purification of Palmitoyl-Epigallocatechin Gallate by Preparative Liquid Chromatography

The acylation reaction product (150 mg) was dissolved in 19 mL of an acetonitrile/LC-MS water mixture (80:20, *v/v*) and purified by preparative HPLC/UV-*vis* (Model Agilent Infinity II equipped with an Agilent 1290 Infinity II fraction collector, Agilent Technologies, Waldbronn, Germany). The column used for the chromatographic resolution was an Agilent Prep-C18 Scalar (4.6 × 250 mm, 10 μm, Agilent Technologies, Waldbronn, Germany), at room temperature, using LC-MS water and acetonitrile as mobile phases A and B, respectively. The flow rate and injection volume were 1 mL/min and 400 μL, respectively. The target lipophenolic was resolved chromatographically using the following elution gradient (time, %B): (0 min, 5%), (6 min, 10%), (12 min, 15%), (18 min, 20%), (24 min, 50%), (30 min, 80%), and (40 min, 90%), returning to the initial conditions at 50 min. The PEGCG was monitored resorting to UV-detection at 275 nm, which provides the maximum absorption of EGCG and ester derivatives, and the collection was initiated automatically. All fractions collected throughout 5 independent runs were pooled. Then, the solvent was removed by rotary evaporation at 25 °C and analyzed by UHPLC-ESI-QqQ-MS/MS, UHPLC-ESI-Q-ToF-MSn, and NMR to evaluate the efficiency of the purification process and to comprehensively identify the synthesized PEGCG.

### 4.4 Identification of Acylation Reaction Products by Liquid Chromatography Coupled to a Mass Spectrometry Detector

The esterification reaction products were identified with an HPLC 1200 series model (Agilent Technologies, Waldbronn, Germany) equipped with a diode array detector (DAD) and a Bruker HCT Ultra ion trap mass detector (Bruker Daltonics, Bremen, Germany) in series. A Luna C18 column (250.0 × 4.6 mm, 5.0 μm particle size; Phenomenex, Torrance, CA, USA) was used, and the mobile phases and gradients were described in subsection 2.3. The flow rate and injection volume were 800 and 20 μL, respectively. Spectral data were accumulated in the 240–330 nm range, and the chromatograms were analyzed at 275 nm. Nitrogen was used as the nebulizing gas at a pressure of 65.0 psi, and the flow rate was adjusted to 11 L/min. The ionization conditions were fine-tuned to 350 °C and 4.5 kV for capillary temperature and voltage, respectively. The full-scan mass covered the range from *m*/*z* 100 to 1200. Collision-induced fragmentation experiments were performed using helium as the collision gas in the ion trap, with voltage ramping cycles from 0.3 to 2.0 eV. The MS data were acquired in the negative ionization mode, and MS­(n) was carried out automatically on the most abundant fragment ion in MS­(*n* – 1). The acylation reaction products were monitored, and the synthesized PEGCG was identified by comparing its mass fragments, elution order, and UV–vis spectra with those provided by the authentic standard of EGCG.

### 4.5 Identification of Purified Palmitoyl-Epigallocatechin Gallate by Liquid Chromatography Coupled to Mass Spectrometry and Nuclear Magnetic Resonance

To confirm the identity of the synthesized and purified PEGCG, the extracts obtained were assessed by resorting to LC coupled with time-of-flight (ToF) and triple quadrupole (QqQ) tandem mass spectrometers. The exact mass analysis was performed using an Acquity UPLC I-Class system (Waters Corporation, Milford, USA) and a C18 column measuring 2.1× 50 mm, 1.7 μm (Waters, Milford, CT, USA), at 40 °C. The flow rate and injection volume were 0.3 mL/min and 7 μL, respectively. Mobile phases A and B consisted of LC-MS water/formic acid (99.9:0.1, *v/v*) and acetonitrile, respectively. The chromatographic separation of PEGCG was carried out by applying the following gradient (time, %B): (0 min, 30%); (2.00 min, 95%); (6.00 min, 98%); and (6.50 min, 30%), returning to the initial conditions at 8.50 min with 2 min of post-time equilibration. The UPLC system was coupled to a quadrupole time-of-flight (QToF) maXis impact mass spectrometer with a resolution of ≥55 000 fwhm (Bruker Daltonics, Bremen, Germany). The MS acquisition was performed in a scan range from *m*/*z* 50 to 1200. The MS assessment was conducted using HR-QToF-MS in ESI-positive mode with broadband collision-induced dissociation. The collision energy and the capillary voltage for the ESI-positive mode were set up at 24 eV and 4.5 kV, respectively. Nitrogen was used as the desolvation and nebulizing gas, with a flux of 9.00 L/min and 2.00 bar, respectively. The drying temperature was set to 200 °C. The external calibrant solution was delivered by a KNAUER Smartline Pump 100 with a pressure sensor (KNAUER, Berlin, Germany). The instrument was calibrated externally before each sequence with a 10 mM sodium formate solution. The mixture was prepared by adding 0.50 mL of formic acid and 1.00 mL of 1.00 M sodium hydroxide to an isopropanol/LC-MS water solution (1:1, *v/v*).

Additionally, for the selective confirmation of the PEGCG identity in the synthesized and purified fraction, its fragments were assessed in the negative mode using an ultrahigh-performance liquid chromatograph (UHPLC) coupled with a 6460 triple quadrupole MS/MS (UHPLC-QqQ-MS/MS) (Agilent Technologies, Waldbronn, Germany), using the analytical column ACQUITY BEH C18 1.7 μm (2.1 × 50 mm) (Waters, Milford, CT, USA), according to the methodology previously validated for the analytical resolution of other lipophenols.^25^ The mobile phases A and B consisted of HPLC–MS grade water/formic acid (99.9:0.1, *v/v*) and acetonitrile, respectively. The flow rate and injection volume were 0.3 mL/min and 15 μL, respectively. The chromatographic resolution of PEGCG was achieved using the following linear gradient (time (min), % B): (0.00, 30%); (3.00, 98%); and (6.00, 100%), returning to the initial conditions at 6.10 min with 1.5 min of post-time equilibration. The identification and quantification of the target ester derivative were achieved by mass spectrometry operated in MRM mode, resorting to retention time and fragmentation patterns, and recording quantification and confirmation transitions set up for PEGCG. Data acquisition and processing were performed using MassHunter software, version B.08.00 (Agilent Technologies, Waldbronn, Germany).

The structure of PEGCG was further assessed by ^1^H and ^13^C NMR spectra that were recorded at the Edificio Bioincubadora of the Faculty of Pharmacy, University of Castilla-La Mancha, on a 400 MHz Varian Inova NMR spectrometer (California, USA) with the probe controlled at 25 °C. The purified fraction was concentrated under reduced pressure, dissolved in 0.4 mL of acetone-*d*_6_ (^1^H NMR) and methanol-*d*_4_ (^13^C NMR), and placed in a 5 mm NMR tube. Chemical shifts are expressed as parts per million (ppm, δ) and are referenced to solvent signals (^1^H/^13^C): C_3_D_6_O (acetone-*d*_6_) (2.05/29.84 ppm) and CD_3_OD (methanol-*d*_4_) (4.87 and 3.31/49.00 ppm) were used as internal standards. Signal descriptions include: s = singlet, d = doublet, t = triplet, and m = multiplet. All coupling constants are absolute values, and *J* values are expressed in Hertz (Hz).

### 4.6 Molecular Docking Computationally Performed by Deep Learning

A blind molecular docking procedure must be developed to locate feasible ligand conformations and attachment spots in the protein combinations across the entire receptor. The tool selected for this task was DiffDock,^44^ which employs diffusion generative models based on neural networks to search for those binding pockets. The atom coordinates obtained from this process were then entered into the molecular docking software, GNINA,^45^ to predict binding energy and affinity values, score the quality of the pose obtained by GNINA, and locate it in the pocket proposed by DiffDock. In this context, it has to be highlighted that GNINA is a fork of SMINA^46^ and AutoDock Vina,^47^ incorporating different ensembles of convolutional neural networks (CNNs) as scoring functions. The enhanced utility of these scoring functions, CNNscore and CNNaffinity, lies in their capacity to retrieve extra evidence of feasible protein–ligand interactions beyond that of their predecessors. The pose quality score (CNNscore) is a metric that ranks the optimal docking poses based on pretrained models developed through deep learning from extensive data sets of known receptor–ligand complexes, considering factors such as shape complementarity, electrostatic interactions, and hydrophobic effects. On the other hand, CNNaffinity estimates the strength of the interaction, expressed in the logarithm (Ln) of Units/mol. In this study, the pipeline DiffDock-GNINA for molecular docking was used to determine the binding affinity of EGCG vs PEGCG against the human COX-2 enzyme (P35354; prostaglandin G/H synthase 2, provided by SWISS-MODEL,^48^ available at https://swissmodel.expasy.org/repository/67cfdacdfc0185afce3b880d/report, accessed on May 1, 2025) using DiffDock^44^ and GNINA molecular docking software (version 1.3; https://github.com/gnina/gnina).^48^

### 4.7 Cell Lines, Culture Conditions, and Cell Viability Tests

The human colon adenocarcinoma (Caco-2) cell line (ATCC HTB-37) was obtained from the American Type Culture Collection (ATCC) (Rockville, MD, USA). Cultures were grown at 37 °C with 5% CO_2_ and 70% humidity in Eagle’s minimal essential medium (EMEM) supplemented with 10% fetal bovine serum (FBS), 1% (*v/v*) nonessential amino acids, 2 mM l-glutamine, and 100 U/mL penicillin/streptomycin. Cells were maintained in a monolayer below 80% confluence until different experimental procedures.

The cytotoxicity of EGCG and synthesized PEGCG was determined in vitro on Caco-2 cells (passages 17 and 20) by developing the trypan blue exclusion assay. For this, growing cells were seeded into a 24-well tissue culture plate (Nunc, Roskilde, Denmark) at 10^5^ cells/well density. After 24 h, the growth medium was replaced by applying decreasing concentrations of EGCG and PEGCG (from 1.000 to 0.001 μmol/L). Following 24 and 48 h of incubation, cells were trypsinized, stained with trypan blue, and counted using a hemocytometer.

### 4.8 Quantitative Determination of Human Cyclooxygenase-2 Expression and Activity in Intestinal Caco-2 Cells

The cell culture supernatants were evaluated for in vitro human cyclooxygenase-2 (COX-2) expression with a specific enzyme-linked immunosorbent assay (ELISA) kit, according to the manufacturer’s instructions (ab267646, Abcam, Cambridge, UK). Briefly, Caco-2 cells were exposed to a proinflammatory stimulus (25 ng/mL IL-1β) for 10 h. Cells that were untreated and those exposed only to IL-1β were considered negative and positive controls, respectively. The capacity of EGCG and PEGCG to modulate the expression of COX-2 was determined by pretreating cells with 1 μM of each compound for 1 h before exposure to the proinflammatory conditions. Then, supernatants were collected, processed according to the instruction manual, and read at 450 nm using an Infinite M200 microplate reader (Tecan, Grödig, Austria) immediately. Results for the COX-2 concentration were expressed as ng/mL.

Additionally, the assessment of the capacity of EGCG compared to PEGCG as inhibitors of COX-2 activity was evaluated with a specific ELISA kit according to the manufacturer’s instructions (Cayman Chemicals, Item No. 701080, Michigan, USA). Briefly, this assay directly measured PFG_2α_ by stannous chloride (SnCl_2_) reduction of COX-derived PGH_2_ produced in the COX reaction. The prostanoid product is quantified using an antiserum specific to PFG_2α_ spectrophotometrically at 412 nm.

### 4.9 Extraction and Quantification of Oxylipin-Markers of Inflammation and Oxidative Stress

For the evaluation of the selected oxylipins, markers of inflammation (the prostaglandins PGF_2α_ and PGE_2_) and oxidative stress (the isoprostane 8-iso-PGF_2α_) produced by Caco-2 cells treated and untreated with the target bioactive compounds under proinflammatory conditions, the supernatants were processed according to the methodology previously described.^49^ Briefly, the supernatants (500 μL) underwent enzymatic hydrolysis for 2 h at 37 °C using β-glucuronidase-sulfatase (glucuronidase activity (>85 000 units/mL) and sulfatase activity (≤7500 units/mL)). Afterward, 625 μL of 200 mM HCl in methanol was added, and the samples were centrifuged at 11 000 *g* for 5 min at 4 °C. The supernatants were cleaned up by SPE using the Strata X-AW cartridges, applying the methodology described by Medina et al.^32^ Subsequently, the PGF_2α_, PGE_2_, and 8-iso-PGF_2α_ concentrations were analyzed using UHPLC-ESI-QqQ-MS/MS (Agilent Technologies, Waldbronn, Germany).

The chromatographic resolution of oxylipins was achieved by UHPLC-ESI-QqQ-MS/MS, applying the methodology and settings described in the literature^49^ and using an ACQUITY BEH C18 column (2.1 × 150 mm, 1.7 μm pore size) (Waters, MA, USA). The identification and quantification of PGF_2α_, PGE_2_, and 8-iso-PGF_2α_ were attained by analyzing pre-established fragmentation patterns and retention times relative to authentic standards. The spectrometry analysis was performed by MRM, operated in the ESI negative mode, and applying the ion optics settings previously optimized.^32,50^ The isoprostanoids’ concentration was calculated according to standard curves prepared each analysis day. Data acquisition and processing were performed using MassHunter software version B.08.00 (Agilent Technologies, Waldbronn, Germany).

### 4.10 Statistical Analysis

All analyses were performed in triplicate (*n* = 3), and the data were expressed as the mean ± SD. Statistical tests were performed at a 5% significance level using the SPSS 29.0 software package (LEAD Technologies, Inc., Chicago, USA). Data were subjected to a one-way analysis of variance (ANOVA) after confirming that the ANOVA requirements regarding the normal distribution of the residuals and the homogeneity of variance were met through the Kolmogorov–Smirnov and Levene tests, respectively. When statistical differences were identified, the variables were compared using Tukey’s multiple range test.

## Supplementary Material



## Data Availability

The data of this article have been included in this manuscript.

## Notes

^1^H and ^13^C NMR spectra of the purified PEGCG and 3D visualisation of the molecular docking results (PDF)
