# Supplementary material for: Palmitoyl-Epigallocatechin Gallate Modulates COX-2-Based Production of Inflammation-Related Oxylipins: Synthesis, Characterization, and Bioevaluation In Vitro and In Silico
Source: ACS Omega. 2025 Jul 29;10(31):34917–29. doi: 10.1021/acsomega.5c04117 (PMC12355247; doi:10.1021/acsomega.5c04117)

Research article

Palmitoyl-epigallocatechin gallate modulates COX-2-based production of inflammation-related oxylipins: Synthesis, Characterisation, and Bioevaluation *in vitro* and *in silico*

Concepción Medrano-Padial[a,†], Pablo Fuentes-Soriano[b,†], Diego Hernández-Prieto[a], Cristina García-Viguera[a], Raúl Domínguez-Perles[a,*], Carlos Romero-Nieto[b,*], Sonia Medina[a]

[a] *Laboratorio de Fitoquímica y Alimentos Saludables (LabFAS), CSIC, CEBAS, Campus Universitario de Espinardo, Edificio 25, 30100 Murcia, Spain.*

[b] *Faculty of Pharmacy, University of Castilla-La Mancha, Calle Almansa 14 – Edif. Bioincubadora, 02008 Albacete, Spain*

* Corresponding author: Laboratorio de Fitoquímica y Alimentos Saludables (LabFAS), CSIC, CEBAS, Campus Universitario de Espinardo, Edificio 25, 30100 Murcia, Spain. *E-mail address*: rdperles@cebas.csic.es (R. Domínguez-Perles) and Faculty of Pharmacy, University of Castilla-La Mancha, Calle Almansa 14 – Edif. Bioincubadora, 02008 Albacete, Spain. *E-mail address*: carlos.romeronieto@uclm.es (C. Romero-Nieto).

[†] These two authors contributed equally to the present work.

SUPPLEMENTARY FILES

Supplementary Figure 1. ^{1}H and ^{13}C NMR spectra of the purified PEGCG.

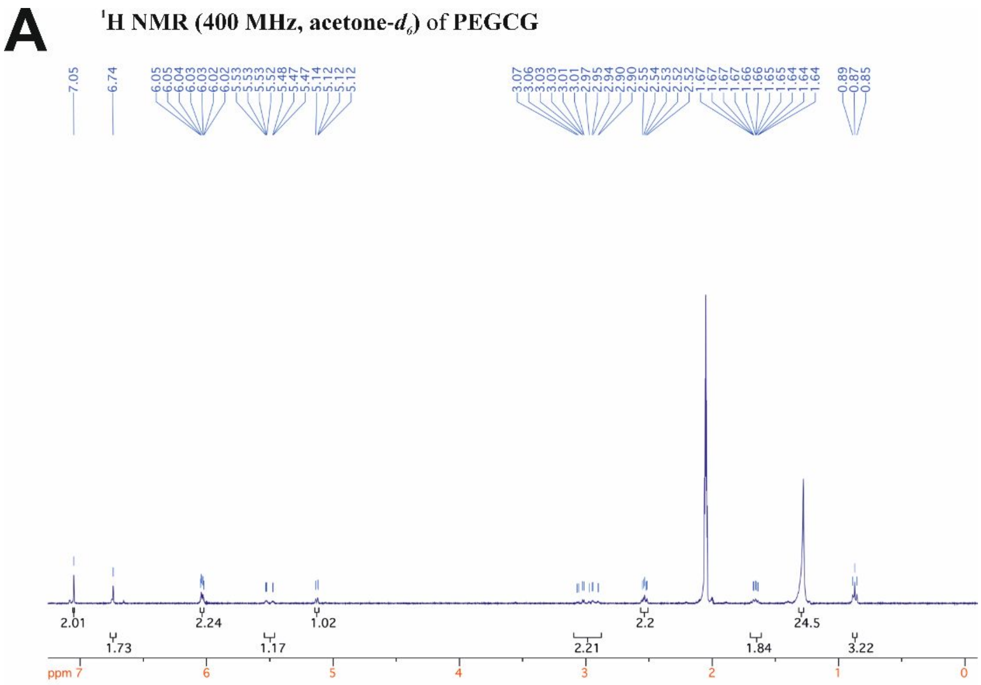

^{1}H NMR (400 MHz, acetone-d_6): δ 7.05 (s, 2H), 6.74 (s, 2H), 6.05 – 6.02 (m, 2H), 5.50 (d, 1H), 5.13 (d, 1H), 3.07 – 2.90 (m, 2H), 2.54 (td, 2H), 1.65 (m, 2H), 1.28 (s, 24H), 0.87 (t, 3H).

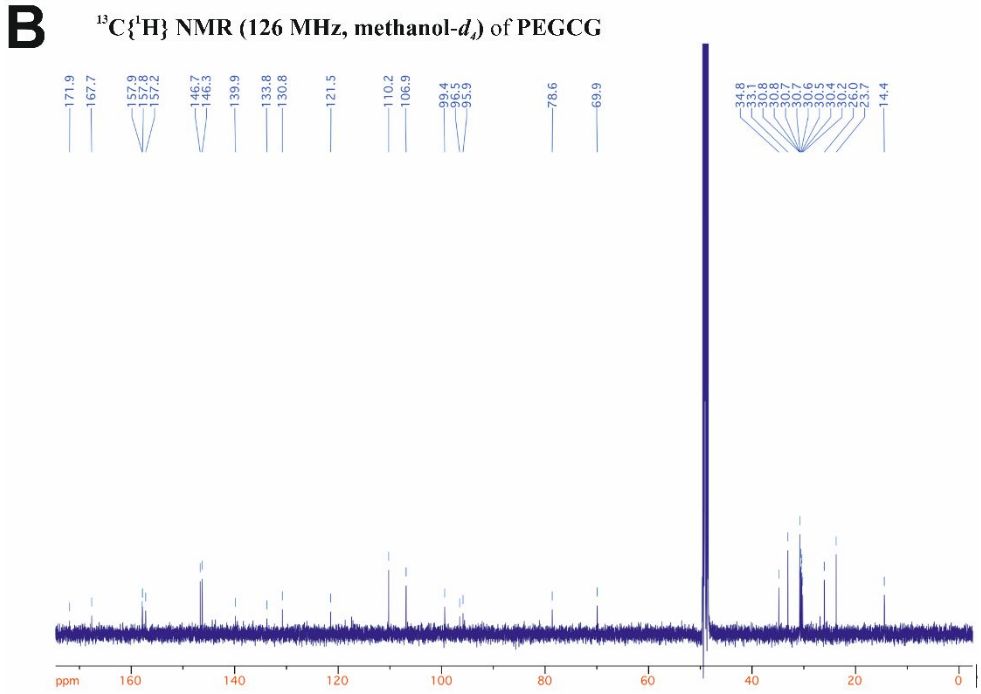

^{13}C NMR (126 MHz; methanol-d_4): δ 171.9, 167.7, 157.9, 157.8, 157.2, 146.7, 146.3, 139.9, 133.8, 130.8, 121.5, 110.2, 106.9, 99.4, 96.5, 95.9, 78.6, 69.9, 34.8, 33.1, 30.8, 30.8, 30.7, 30.7, 30.7, 30.6, 30.5, 30.4, 30.2, 26.8, 26.0, 23.7, 14.4.

Supplementary Figure 2. 3D visualizations of the molecular docking results. Ranked conformations were provided by blind docking performed by DiffDock, and the top three calculations for each ranked conformation were carried out by GNINA. (A) Rank 1 conformation of EGCG in pink and further calculations in purple; (B) Rank 2 conformation of EGCG in white and further calculations green; (C) Rank 3 conformation of EGCG in dark pink and further calculations in light blue; (D) Rank 1 conformation of PEGCG in light blue and further calculations in dark blue; (E) Rank 2 conformation of PEGCG in green and further calculations in different colors; (F) Rank 3 conformation of PEGCG in light green and further calculations in light blue.

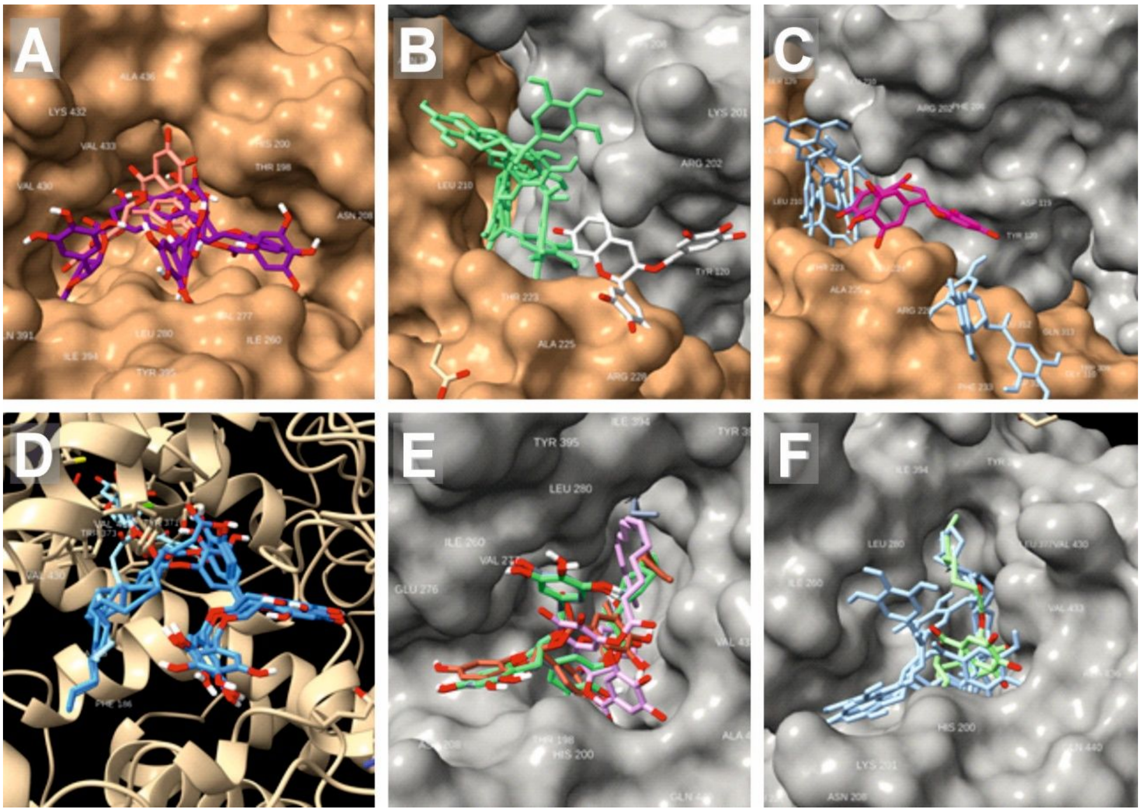

Supplement: Supplementary file 1 [file ao5c04117_si_001.pdf]
